# Hémophagocytose et coagulation intravasculaire disséminée au cours de la leishmaniose viscérale de l'adulte: trois nouveaux cas

**DOI:** 10.11604/pamj.2015.22.96.5662

**Published:** 2015-10-01

**Authors:** Imène Boukhris, Samira Azzabi, Eya Chérif, Ines Kéchaou, Sonia Mahjoub, Chékib Kooli, Karim Aoun, Narjes Khalfallah

**Affiliations:** 1 Service de Médecine Interne B, Hôpital Charles Nicolle, Faculté de Médecine de Tunis, 1006 Bab Saadoun, Université de Tunis El Manar, Tunis, Tunisie; 2 Laboratoire d'Hématologie, Hôpital la Rabta, Tunis, Tunisie; 3 Laboratoire de Parasitologie, Institut Pasteur de Tunis, Tunis, Tunisie

**Keywords:** Hémophagocytose, coagulation intravasculaire disséminée, leishmaniose viscérale de l′adulte, hemophagocytosis, disseminated intravascular coagulation, visceral leishmaniasis

## Abstract

Les atteintes cliniques et biologiques communes au syndrome d'activation macrophagique (SAM) et à la leishmaniose viscérale (LV) rendent le diagnostic étiologique du SAM très difficile. Cette association est rare et grave. Nous rapportons trois nouvelles observations de SAM secondaire à une LV, compliquées de coagulation intravasculaire disséminée (CIVD). Il s'agissait de trois hommes, âgés respectivement de 31, 20 et 60 ans. Le tableau était fait de fièvre et de splénomégalie associés à une pancytopénie et une CIVD. Le diagnostic de LV était fait par le myélogramme, les sérologies et la polymerase chain reaction. Chez l'un de nos patients, une deuxième sérologie était nécessaire. Tous nos patients étaient traités par Glucantime^®^ avec une bonne évolution. Un cas de pancréatite aigue était noté. En en zones d'endémie, devant un SAM compliqué de CIVD, une LV doit être recherchée, en répétant si nécessaire certaines explorations initialement négatives. Le pronostic dépend de la rapidité du traitement spécifique.

## Introduction

La leishmaniose viscérale (LV) de l'adulte est une cause rare du syndrome d'activation macrophagique (SAM). Les atteintes communes de la LV et du SAM rendent le diagnostic étiologique très difficile en cas de SAM [1, 2]. Les troubles de l'hémostase secondaires à une LV ou à un SAM sont fréquents, mais la survenue d'une coagulation intra-vasculaire disséminée (CIVD) est peu rapportée [3, 4]. Le traitement de la LV par Glucantime^®^ peut être responsable de plusieurs effets indésirables; la survenue d'une pancréatite est exceptionnelle [5]. Nous rapportons trois observations de SAM avec CIVD, secondaires à une LV de l'adulte traités par Glucantime^®^, avec survenue d'une pancréatite chez l'un des patients. Le diagnostic de SAM et de CIVD étaient respectivement retenu selon les cirières modifiées de Henter (Tableau 1) [6] et le score diagnostic «Japanese Association for Acute Medicine » (JAAM) (Tableau 2) [7].

**Tableau 1:** Critères diagnostiques du syndrome d'activation macrophagique [6]

Au moins cinq critères parmi les suivants:
Ø Fièvre
Ø Splénomégalie
Ø Cytopénies affectant au moins deux lignées
o Hémoglobine < 9 g/dL o Plaquettes < 100000/mm^3^ o Polynucléaires neutrophiles < 1000/mm^3^
Ø Hypertriglycéridémie et/ou hypofibrinogénémie
o Triglycérides >2,60 g/l o Fibrinogène < 1,5 g/L
Ø Hémophagocytose dans la moelle osseuse, la rate ou les ganglions lymphatiques
Ø Ferritinémie supérieure à 500 µg/L
Ø Activité des cellules Natural Killer basse ou nulle (selon les références du laboratoire local)
Ø Récepteur soluble à l'IL-2 supérieure à 2400UI/ml

**Tableau 2:** Score diagnostique de la CIVD selon la Japanese Association for Acute Medicine (JAAM) [7]

Critères de réponse inflammatoire systémique	
≥3	1
0-2	0
Numération plaquettaire (G/L)	
< 80 ou diminution > 50% en 24 heures	3
≥80 et < 120 ou diminution > 30% en 24 heures	1
≥120	0
Fibrinogène (g/L)	
< 3,5	1
≥3,5	0
Temps de Quick (ratio patient/témoin)	
≥1,2	1
< 1,2	0
Produits de dégradation de la fibrine (mg/L)	
≥25	3
≥10 et < 25	1
<10	0
CIVD si le score est ≥5 points.	

## Patient et observation

Observation 1: un patient âgé de 31 ans, était hospitalisé pour une fièvre depuis 15 jours, associée à une asthénie, une anorexie et un amaigrissement. L'examen trouvait une température à 39°C, un état général altéré, une pâleur cutanéo-muqueuse, et une hépato-splénomégalie. Les paramètres biologiques sont notés dans le Tableau 3. La ponction sternale montrait une hémophagocytose. Devant la pancytopénie, l'hypertriglycéridémie, l'hémophagocytose, la fièvre et la splénomégalie, le diagnostic de SAM était retenu (5 critères de Henter). Au bilan d'hémostase (Tableau 3), il y avait une CIVD avec un score de JAAM à 9 points. L'examen direct du frottis de la ponction sternale montrait deux corpuscules évoquant des corps de leishmanies. La sérologie de la leishmaniose était positive à 1/1600 en IFI. D'autres examens ont été pratiqués, ils étaient tous négatifs (Tableau 4). Le patient était traité par Glucantime en intramusculaire à la dose de 60 mg/kg/j, pendant 21 jours. L’évolution était favorable avec un recul actuel de 7 ans.

**Tableau 3:** Les données biologiques des trois patients

Observations	Observation 1	Observation 2	Observation 3
**Hémogramme**	GB = 1500 EB/mm^3^	GB = 2400 EB/mm^3^	GB = 2050 EB/mm^3^
	HB = 7 g/dl	HB = 6,2 g/dl	HB = 10,6 g/dl
	PLQ = 62000/mm^3^	PLQ = 67000/mm^3^	PLQ = 46000/mm^3^
	Rétic = 49000/mm^3^	Rétic = 50400/mm^3^	Rétic = 41400/mm^3^
**VS**	127 mm à H1	75 mm à H1	63 mm à H1
**CRP**	293mg/l	104mg/l	142 mg/l
γ**globulines**	26g/l	21g/l	18g/l
**Ferritine**	Dosage non fait	1000*nle	50*nle
**Triglycérides**	3,89 g/l	2,19 g/l	6,29 g/l
**LDH**	15*nle	11*nle	2,5*nle
**Bilan hépatique**	ASAT: 5*nle	ASAT = 4,5*nle	ASAT :nle
	ALAT 2,5*nle	ALAT = 2,5*nle	ALAT :nle
	γGT:3*nle	γGT:2*nle	γGT : 4*nle
	PAL: nle	PAL: nle	PAL: nle
**Bilan d'hémostase**	TP:53%	TP:23%	TP:70%
	Rapport M/T (TCA):1,6	Rapport M/T (TCA) 〉2	Rapport M/T (TCA):1,2
	D-Dimères: 50*nle	D-Dimères: 30*nle	D-Dimères: 25*nle
	Fibrinogène: 2,59 g/l	Fibrinogène: 0,5 g/l	Fibrinogène: 0,6 g/l

GB: globules blancs, EB: éléments blancs, HB: hémoglobine, PLQ: plaquettes, Rétic: réticulocytes, VS: vitesse de sédimentation, CRP: C réactive protéine, Rapport M/T: rapport malde/témoin, TP: temps de prothrombine, TCA: temps de céphaline activée,TG: triglycérides, LDH: lactate deshydrogénase, αGT: αglutamyl transférase, PAL: phosphatases alcalines, ASAT: aspartate aminotransférase, ALAT: alanine aminotransférase, Nle: valeur normale, Nle: (x) fois la normale.

**Tableau 4:** Explorations chez les trois patients.

Observations	Observation 1	Observation 2	Observation 3
**Bilan infectieux**	Hémocultures (-) ECBU (-)BK crachats (-) BK urines(-) Sérologie HBV(-) Sérologie HCV(-) Sérologie VIH(-) Sérologie Wright(-) Sérologie Widal(-) Sérologie leishmaniose :(+)à 1/1600 en IFI	Hémocultures (-) ECBU (-) BK crachats (-) Coproculture (-)Sérologie HB (-) Sérologie HC (-)Sérologie VIH (-) Sérologie CMV (-) Sérologie EBV (-) Sérologie PB19 (-) Sérologie HSV (-) Sérologie Wright (-) Sérologie Widal (-) 1^ère^ SérologieLeishmaniose (-) 2^ème^ Sérologie leishmaniose (+) à 1/200 en IFIPCR leishmania sp (+)	Hémocultures (-) ECBU (-) BK crachats (-) Sérologie HB (-) Sérologie HC (-) Sérologie VIH (-) Sérologie Wright (-) Sérologie Widal (-) Sérologie leishmaniose(+) 1/400 en IFI PCR leishmania sp (+) à 1,65 p/ml
**Examans d'imagerie**	Radio thorax : nle ETT : nle Echo abdominale : splénomégalie	Radio thorax : nle TDM cérébrale : nle TDM TAP : hépatomégalie Splénomégalie ETT : nle	Radio thorax : nle TDM TAP : Splénomégalie, pancréatite (sous glucantime^®^)
**Autres explorations**	AAN < 0	AAN < 0 BOM : nle Caryotype : nl	AAN < 0

ECBU: examen cytobactériologique des urines, IFI: immunofluorescence indirecte, PCR: polymérase chain reaction,BK: bacille de Kock, HB: hépatite B, HC: hépatite C, VIH: virus de l'immunodéficience humaine, CMV: cytomégalovirus, EBV: Epstein Bar virus, HSV: Herpes Simplex virus, PB19: parvovirus B19, ETT: echocardiographie transthoracique, TDM: tomodensitométrie, TAP: thoraco-abdomino-pelvienne, BOM: biopsie ostéodensitométrie, AAN: antocorps antinucléaires, Nl: normal, (-): résultat négatif.

Observation 2: un patient âgé de 20 ans, était hospitalisé pour une fièvre évoluant depuis 3 mois avec un amaigrissement, une asthénie et des épistaxis. A l'examen le patient était fébrile à 39,5°C, il avait un état général altéré et une hépato-splénomégalie. Les paramètres biologiques sont notés dans le Tableau 3. Le myélogramme montrait une hémophagocytose. Devant la pancytopénie, l'hyperferritinémie, l'hémophagocytose, la fièvre et la splénomégalie, le diagnostic de SAM était retenu (5 critères de Henter). Les prélèvements bactériologiques, les sérologies infectieuses et l'imagerie étaient sans anomalies (Tableau 4). La biopsie médullaire montrait une moelle de richesse normale, sans myélofibrose ni envahissement tumoral. Le caryotype oncologique sur moelle osseuse ne trouvait pas d'anomalies chromosomiques. L’évolution était marquée par l'aggravation de l’état général avec apparition d'une CIVD (score de JAAM à 6 points) compliquée d'un syndrome hémorragique et d'une dégradation des constantes hémodynamiques, nécessitant une prise en charge en réanimation. Une antibiothérapie probabiliste à large spectre était prescrite, associée à un remplissage vasculaire, des transfusions de culots globulaires et de plasma frais congelé. Devant le SAM, sans cause infectieuse identifiée, le patient était traité par une corticothérapie à la dose de 1 mg/kg/j, suivie d'une cure d'immunoglobulines intraveineuse Ig-IV à la dose de 2g/kg, sans amélioration. Les examens d'imagerie et les sérologies initialement négatives, ont été redemandés. La deuxième sérologie de la leishmaniose, était revenue positive à 1/200 en IFI. Ce résultat était confirmé par Polymérase Chain Réaction (PCR), mettant en évidence de l'ADN de Leishmania sp. Un traitement par glucantime^®^ à la dose de 50 mg/kg/j était instauré. L’évolution était favorable avec un recul actuel de 4 ans.

**Figure 1:**
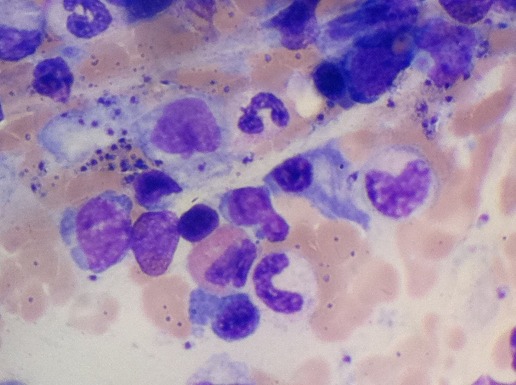
Photo d'un fort grossissement microscopique d'une lame d'un myélogramme montrant des corps de Leishmanies en extra et intracellulaires

**Figure 2:**
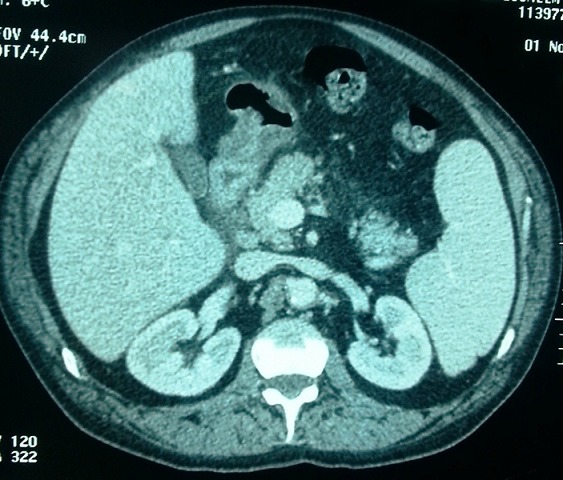
Photo d'une plaque d'une tomodensitométrie abdominale montrant une infiltration de la graisse péri-pancréatique et une densification interpancréatico-duodénale

Observation 3: un patient âgé de 60 ans était hospitalisé pour une fièvre évoluant depuis 10 jours avec une asthénie. A l'examen, il avait une fièvre à 39°C et une splénomégalie. Les paramètres biologiques sont notés dans le Tableau 3. Au bilan d'hémostase, il y avait une CIVD (score de JAAM à 5 points). La ponction sternale mettait en évidence une hémophagocytose, ainsi que la présence de corps de leishmanies (Figure 1). La sérologie de la leishmaniose était positive à 1/400 en IFI, la PCR mettait en évidence de l'ADN de Leishmania sp (charge parasitaire à 1,65 p/ml). Le reste du bilan étiologique était négatif (Tableau 4). Un traitement par glucantime^®^ était administré à la dose de 60mg/kg/j avec introduction progressive. Au huitième jour de traitement, le patient avait présenté des douleurs abdominales avec une ascension des amylases et des lipases respectivement à deux et trois fois la normale. La TDM abdominale montrait une infiltration de la graisse péri-pancréatique et une densification interpancréatico-duodénale (Figure 2). Le canal de Wirsung n’était pas dilaté, la voie biliaire principale était non distendue et les voies biliaires intra et extra-hépatiques étaient fines. Le diagnostic de pancréatite aigue était retenu. Le patient n'avait pas d'antécédents d'alcoolisme, de traumatisme, ni de pathologie vésiculaire lithiasique. La calcémie était normale. L'aspect radiologique du pancréas n’était pas tumoral. L'hypertriglycéridémie était récente. Le Glucantime^®^ était incriminé et arrêté. L’évolution était faite d'une amélioration clinique avec normalisation des enzymes pancréatiques. Après une fenêtre thérapeutique de 7 jours, le Glucantime^®^ a été réintroduit progressivement avec une surveillance enzymatique. Le patient a totalisé 15 jours de Glucantime^®^. L’évolution était favorable. La TDM abdominale de contrôle était sans anomalie. Le contrôle de la sérologie et la PCR de la leishmaniose était négatif. Le recul actuel est de 12 mois.

## Discussion

Le SAM est caractérisé par un ensemble de signes cliniques et biologiques non spécifiques. Le tableau clinique est classiquement fait d'une fièvre, d'une altération de l’état général avec une splénomégalie. Un ictère, une hépatomégalie et des adénopathies sont fréquents [1, 3, 6]. La LV est caractérisée par l'association d'une organomégalie, de fièvre, de cachexie, de pancytopénie et d'hypergammaglobulinémie polyclonale [2, 4, 8]. Les atteintes communes au SAM et à la LV rendent le diagnostic de LV, en cas de SAM, très difficile avec un retard diagnostique et thérapeutique [8]. La LV chez l'adulte immunocompétent est rare, une forte prévalence est notée chez l'adulte infecté par le VIH [9]. Tous nos patients avaient une sérologie VIH négative. En cas de LV, les complications hématologiques sont fréquentes, mais la survenue d'un authentique SAM est rare [2]. Chez nos trois patients, le bilan étiologique à la recherche d'une autre cause au SAM était négatif (Tableau 4). Le diagnostic de certitude de LV reste parasitologique. L'aspiration médullaire par ponction sternale est l'examen le plus utilisé. La sensibilité de l'examen direct sur moelle osseuse varie entre 75 à 99%. Chez nos patients, cet examen était rentable dans deux cas. La culture sur moelle osseuse, peut être positive dans 60 à 100% des cas. La sérologie de la LV est un bon outil diagnostic en absence d'immunodépression, mais la PCR a une meilleure sensibilité, sa spécificité est proche de 100% [8]. La PCR, pratiquée chez deux de nos patients était positive. Le diagnostic de LV est souvent difficile à la phase initiale du SAM. Le clinicien doit rechercher cette étiologie en répétant au besoin, certains examens initialement négatifs, notamment le myélogramme et la sérologie [2], comme c’était le cas dans notre deuxième observation. Au cours de la LV, la survenue d'une CIVD est rarement rapportée [4]. L'imputabilité de la LV dans la survenue de la CIVD était difficile à établir chez nos patients, étant donné la présence du SAM. En cas de SAM, les troubles de l'hémostase sont classiques. Mais notons que même en cas de SAM, la CIVD est rarement rapportée. La CIVD constitue un facteur de mauvais pronostic du SAM vu le risque de complications hémorragiques pouvant être fatales [3, 6]. Dans ce travail, l’évolution était favorable dans tous les cas, malgré la présence de CIVD. Cette bonne évolution était probablement expliquée par le traitement étiologique qui avait permis chez les trois patients le contrôle aussi bien de l'infection que du SAM et de la CIVD. La prise en charge des SAM d'origine infectieuse repose essentiellement sur le traitement de l'agent infectieux, le traitement des défaillances d'organes et le traitement immuno-modulateur de l'hémophagocytose reposant sur les corticoïdes et l’étoposide. L'administration d'immunoglobulines polyvalentes n'est actuellement pas recommandée dans le SAM infectieux, mais quelques succès ont été notés [1, 2]. Dans notre deuxième observation, les immunoglobulines étaient indiquées devant la gravité du tableau. Le traitement de référence de la LV est l'amphotéricine B liposomale. Les dérivés de l'antimoine demeurent le traitement de référence dans de nombreux pays [10]. La tolérance au Glucantime^®^ est variable. Deux tableaux sont classiquement décrits: la stibiointolérance et la stibiointoxication. Dans la troisième observation, nous avons décrit la survenue d'une pancréatite aigue suite à la prescription de Glucantime^®^. La toxicité pancréatique du Glucantime^®^ est exceptionnellement rapportée [5].

## Conclusion

Le SAM secondaire à une LV est rarement rapporté, les atteintes communes au SAM et à la LV rendent le diagnostic étiologique du SAM très difficile. Devant un tableau de SAM secondaire à une LV, les troubles de l'hémostase peuvent évoluer vers une CIVD. Le traitement spécifique de l'agent infectieux s'impose dès que ce dernier a été identifié pour améliorer le pronostic. Nos trois observations se distinguent par l'association de SAM et de CIVD chez des patients présentant une LV de l'adulte. L’évolution sous traitement étiologique était favorable dans les trois cas, malgré la gravité des tableaux de présentation clinico-biologique. Nous avons aussi décrit une observation originale de pancréatite secondaire au traitement par Glucantime^®^. Notre travail mérite d’être poursuivi par une étude prospective et multicentrique, portant sur les complications hématologiques de la LV de l'adulte.

## References

[1] Gonzalez F, Vincent F, Cohen Y (2009). Syndrome d'activation macrophagique d'origine infectieuse: étiologies et prise en charge. Réanimation..

[2] Rajagopala S, Dutta U, Chandra KS, Bhatia P, Varma N, Kochhar R (2008). Visceral leishmaniasis associated hemophagocytic lymphohistiocytosis: case report and systematic review. J Infect..

[3] Créput C, Galicier L, Oksenhendler E, Azoulay E (2005). Syndrome d'activation lymphohistiocytaire: revue de la littérature, implications en réanimation. Réanimation..

[4] Mishra P, Dixit A, Chatterjee T, Bhattacharya M, Bhattacharya J, Dutta P (2004). Disseminated intravascular coagulation as an unusual presentation of Kalaazar: report of two cases. Scand J Infect Dis..

[5] Mc Bride MO, Linney M, Davidson RN, Weber JN (1995). Pancreatitis necrosis following treatment of leishmaniasis with sodium stibiogluconate. Clin infect Dis..

[6] Henter JI, Horne A, Aricó M, Egeler RM, Filipovich AH, Imashuku S (2007). HLH-2004: Diagnostic and therapeutic guidelines for hemophagocytic lymphohistiocytosis. Pediatr Blood Cancer..

[7] Gando S, Saitoh D, Ogura H, Mayumi T, Koseki K, Ikeda T (2009). Disseminated intravascular coagulation (DIC) diagnosed based on the Japanese Association for Acute Medicine criteria is a dependent continuum to overt DIC in patients with sepsis. Thromb Res..

[8] Gatti S, Gramegna M, Klersy C, Madama S, Bruno A, Maserati R (2004). Diagnosis of visceral leishmaniasis: the sensitivities and specificities of traditional methods and a nested PCR assay. Ann Trop Med Parasitol..

[9] Fernández-Guerrero ML, Robles P, Rivas P, Mójer F, Muñíz G, de Górgolas M (2004). Visceral leishmaniasis inimmunocompromised patients with and without AIDS: a comparison of clinical features and prognosis. Acta Trop..

[10] Buffet PA, Rosenthal E, Gangneux JP, Lightburne E, Couppié P, Morizot G (2011). Traitement des leishmanioses en France: proposition d'un référentiel consensuel. Presse Med..

